# Transitioning from hand-sewn to circular stapled cervical anastomosis after minimally invasive esophagectomy: a Western centre propensity score matching analysis

**DOI:** 10.1007/s13304-026-02829-x

**Published:** 2026-08-24

**Authors:** Lorenzo Giorgi, Giovanni Maria Garbarino, Andrea Pansa, Mattia Di Benedetto, Silvia Basato, Salvatore Marano, Rita Alfieri, Carlo Castoro

**Affiliations:** 1 Upper Gastrointestinal Surgery Unit, IRCCS Humanitas Research Hospital, 20089 Rozzano, Milan, Italy; 2 Department of Biomedical Sciences, Humanitas University, 20072 Pieve Emanuele, Milan, Italy; 3 Upper Gastrointestinal Surgery Unit, Ospedale Civile Di Vigevano, 27029 Vigevano, Italy

**Keywords:** Esophageal cancer, Minimally invasive surgery, Anastomotic leak, Propensity score

## Abstract

Cervical esophagogastric anastomosis remains a technically challenging step in esophageal surgery. Hand-sewn techniques have traditionally been favored for their adaptability, while circular stapled anastomosis has gained popularity for its reproducibility and efficiency. However, structured comparative data for cervical anastomosis remain scarce and conflicting. A retrospective monocentric analysis was conducted, based on a prospectively maintained database, on consecutive patients who underwent minimally invasive three-stage esophagectomy (McKeown) with cervical anastomosis for esophageal cancer (January 2018–March 2025). Since February 2022, we have adopted circular stapled cervical anastomosis for all patients, without reverting back. To reduce confounding, propensity score matching was performed using key clinical and oncologic variables. Outcomes of interest included anastomotic leak, anastomotic stricture, postoperative complications, time to oral intake, and hospital length of stay. A total of 127 patients were included, with 36 matched pairs analyzed. The circular stapled group had a lower incidence of anastomotic leak (3/36, 8.3% vs. 7/36, 19.4%) and stricture (6/36, 16.7% vs. 11/36, 30.6%), though differences were not statistically significant. On multivariable analysis, high tumor location was the sole independent predictor of anastomotic leak (OR 3.01, *p* = 0.023), whereas the circular stapled technique demonstrated a non-significant protective trend (OR 0.56, *p* = 0.279). Time to resumption of oral intake was significantly shorter in the stapled group (9 vs. 10 days, *p* = 0.007), and length of hospital stay was reduced (14 vs. 19 days, *p* < 0.001). Overall complication rates and severity were comparable between groups. The transition from hand-sewn to circular stapled cervical anastomosis was feasible, safe, and associated with improved short-term recovery. Although the mechanical approach does not independently reduce leak rates, its high reproducibility and capacity for technical standardization support its role as a valuable strategy for cervical reconstruction following minimally invasive esophagectomy.

**Supplementary Information:**

The online version contains supplementary material available at https://doi.org/10.1007/s13304-026-02829-x.

## Introduction

Esophageal cancer remains a significant global health burden, ranking among the ten most common malignancies worldwide, with over 600,000 new cases and approximately 544,000 deaths reported annually [1]. Despite ongoing advances in multimodal therapy, surgical resection continues to represent the cornerstone of curative treatment for resectable tumors. Reconstruction typically involves the creation of a gastric conduit, with the esophagogastric anastomosis representing the most critical and technically demanding step.

This phase carries substantial risks, particularly anastomotic leaks and strictures, which can adversely affect short-term outcomes, including morbidity and mortality as well as long-term quality of life and overall survival [2, 3].

Cervical esophagogastric anastomosis is commonly employed following subtotal esophagectomy, especially for mid-to-upper thoracic or cervical esophageal tumors, or in select benign conditions. Compared to intrathoracic anastomosis, the cervical approach offers advantages such as wider proximal resection margins for distal esophagus cancers and reduced risk of mediastinal contamination in case of anastomotic leak (AL). However, it is technically challenging due to the anatomical constraints of the neck and the need for a longer gastric conduit to ensure a tension-free reconstruction.

Over the years, various techniques for cervical esophagogastric anastomosis have been described [4]. Despite the paradigm shift brought by minimally invasive platforms in esophageal resection, standardizing the cervical esophagogastric anastomosis remains a significant surgical challenge. Hand-sewn (HS) methods are appreciated for their adaptability to tissue discrepancies and potentially lower stricture rates, whereas stapled techniques are often favoured for their reproducibility and time efficiency, especially in minimally invasive settings, and possibly fewer anastomotic complications [5–8].

This study aims to evaluate our institutional experience during the transition from the hand-sewn technique to an exclusive mechanical circular stapled (CS) approach following minimally invasive three-stage esophagectomy. Specifically, we aimed to analyze whether the implementation of a highly standardized anastomotic model translates into predictable short-term surgical outcomes and accelerated functional recovery.

## Methods

We conducted a retrospective analysis of a prospectively maintained institutional database including patients treated between January 2018 and March 2025. All procedures were performed or directly supervised by a single lead surgeon who established the minimally invasive McKeown esophagectomy program in 2009. The composition of the surgical team and the perioperative protocols remained consistent throughout the entire study period. This study adhered to the Strengthening the Reporting of Observational Studies in Epidemiology (STROBE) reporting guideline (additional information is given in Online Resource 1) and was approved by the Institutional Review Board. All patients had previously provided written informed consent for the research use of clinical data. The study was conducted in accordance with the Declaration of Helsinki and applicable local regulations.

**Fig. 1:**
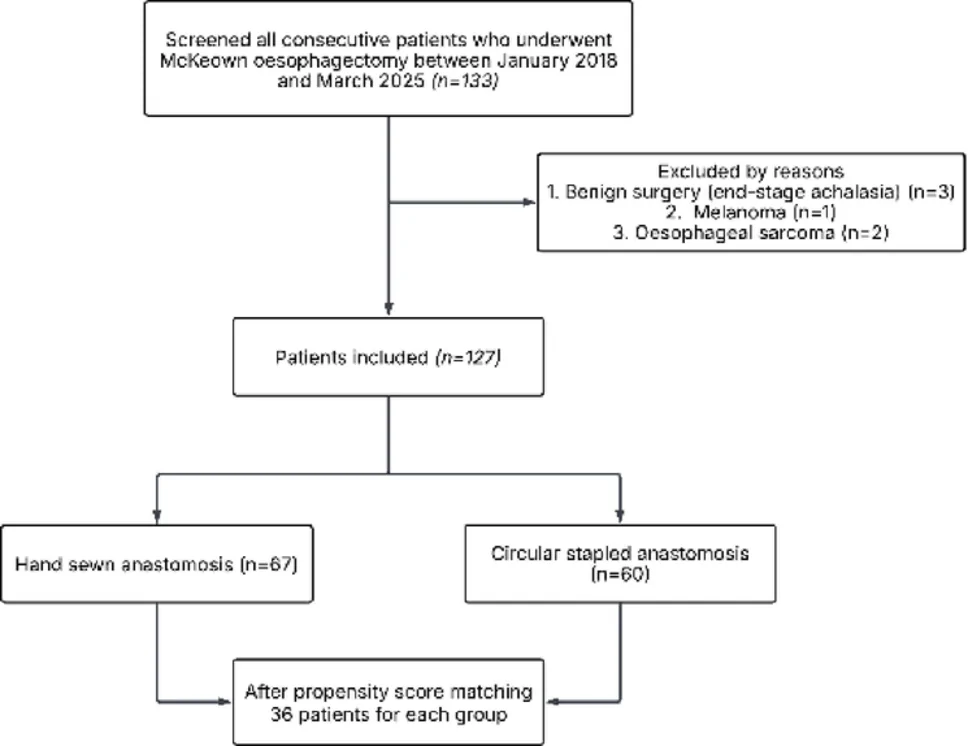
Flowchart of patients meeting inclusion criteria

Patients with benign esophageal conditions or rare histologies not routinely managed with standardized oncologic protocols were excluded to ensure cohort homogeneity regarding resection margin and neoadjuvant therapy (Fig. 1).

Primary endpoints of the study were the incidence of AL and stricture. Secondary endpoints included postoperative complications, length of hospital stay (LOS), and time to oral intake resumption. Given the retrospective nature of this study, the sample size was determined by the inclusion of all consecutive patients meeting the eligibility criteria during the specified study period. While no a priori power calculation was performed, the study intended to analyze the complete institutional experience during the transition from HS to CS cervical anastomosis.

### Surgical treatment and definition of postoperative morbidity

All patients underwent minimally invasive McKeown esophagectomy (thoracoscopy, laparoscopy, left cervical incision) with immediate gastric pull-up reconstruction. Intraoperative indocyanine green fluorescence angiography (ICG-FA) was routinely used to assess gastric conduit perfusion and assist thoracic duct identification. A feeding jejunostomy was placed in all cases.

**Fig. 2:**
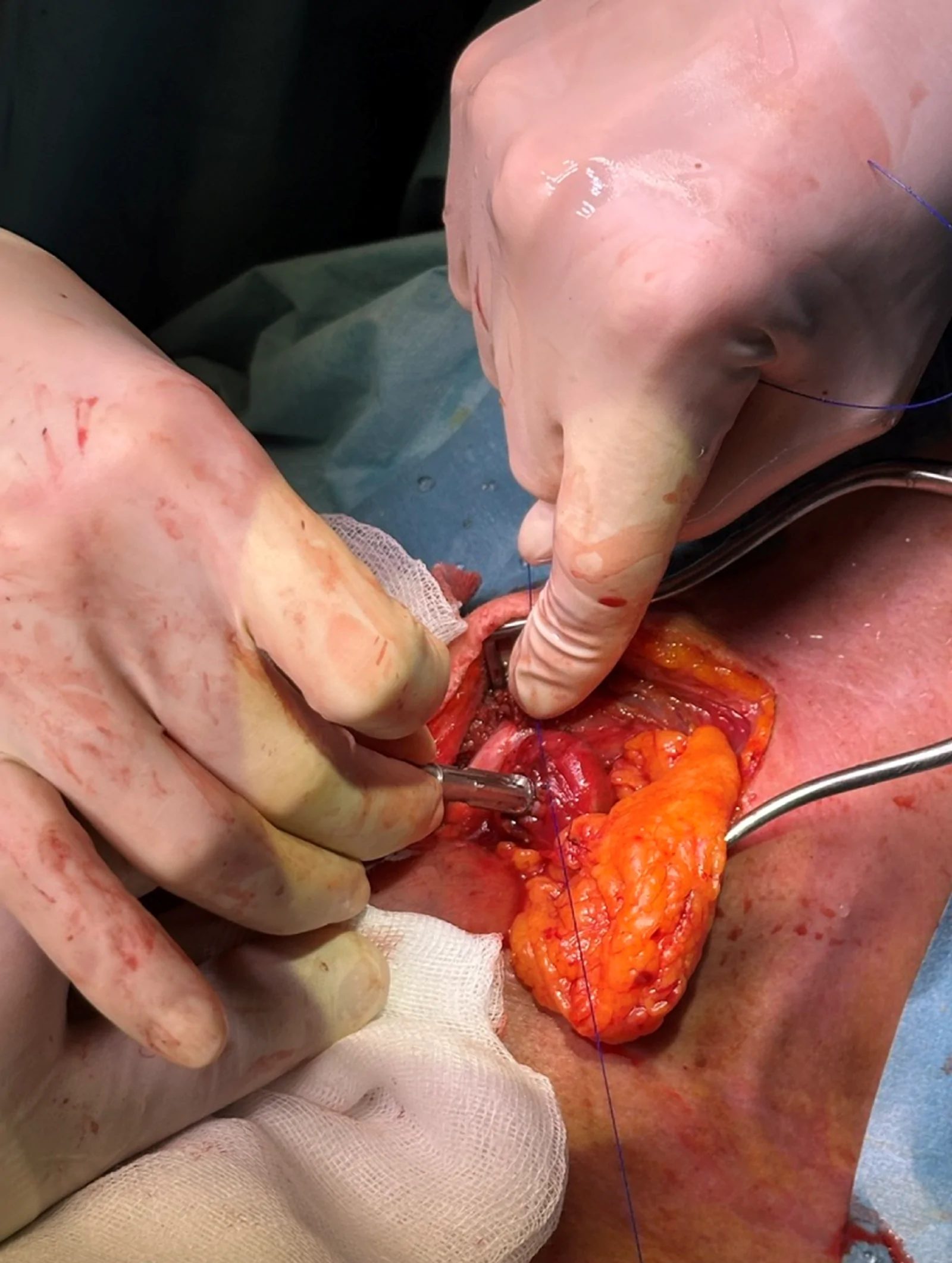
Insertion of the stapler’s anvil and tightening of the Prolene purse-string suture

**Fig. 3:**
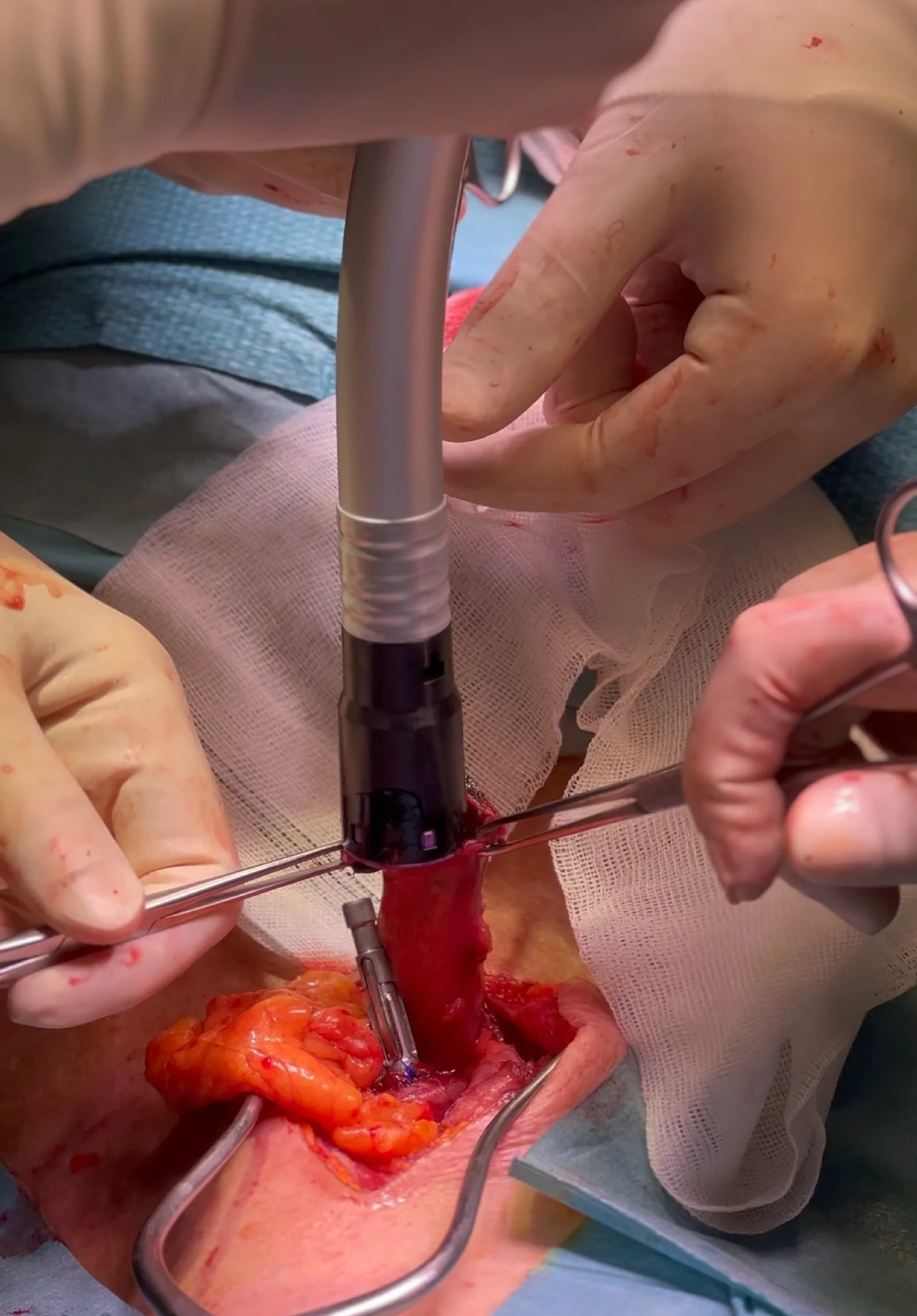
Gastrotomy and insertion of the stapler’s shaft

**Fig. 4:**
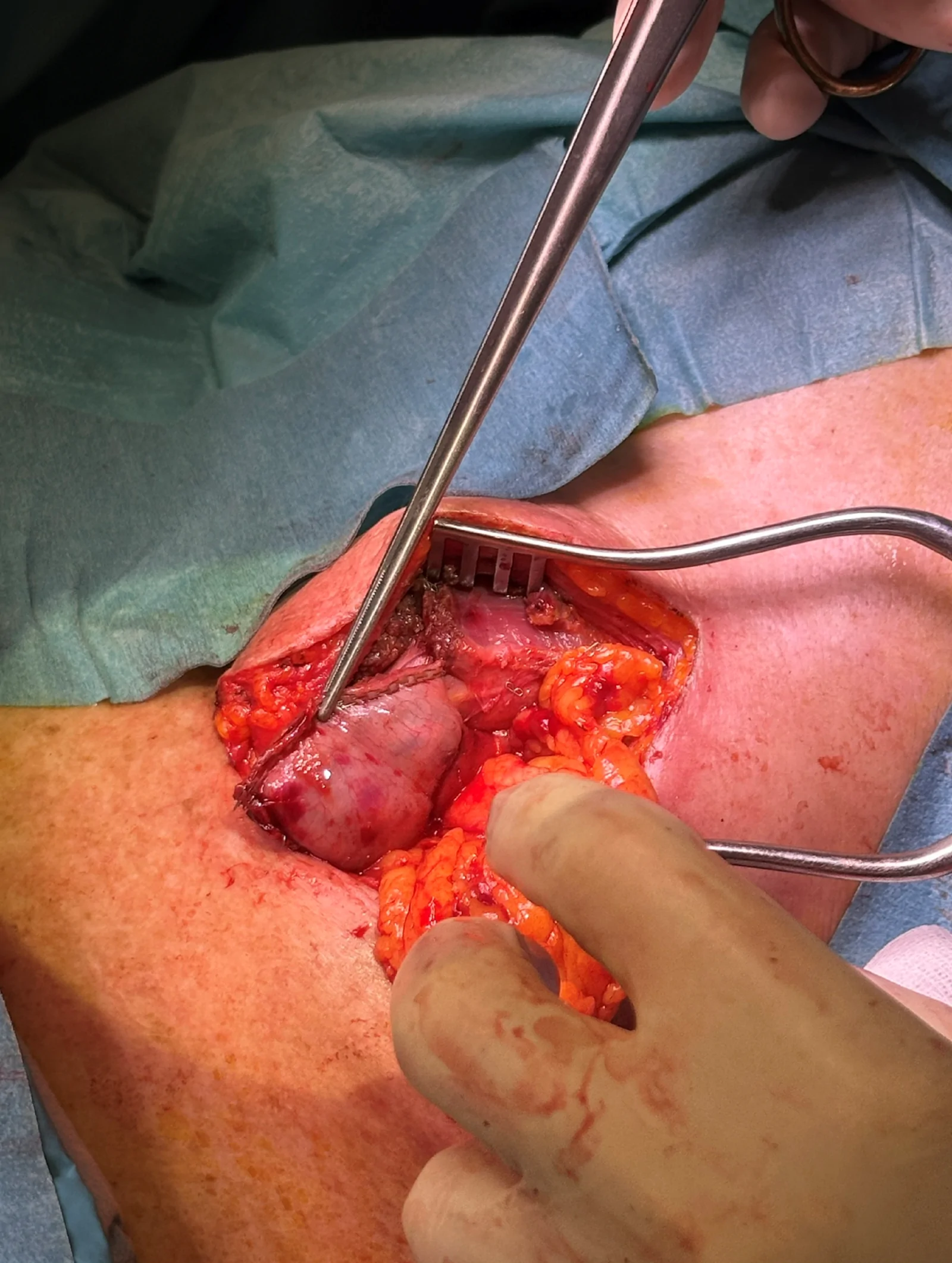
Completed staple line after linear closure

HS anastomoses were performed using a double-layer end-to-side esophagogastric technique: interrupted 4–0 slow-absorbing monofilament sutures for the outer seromuscular layer, and continuous sutures for the mucosal layer. CS end-to-side anastomoses were performed using the ECHELON CIRCULAR™ Powered Stapler (Ethicon, J&J, USA), with a double-layer purse-string suture for the anvil (Fig. 2), anterior gastrotomy for stapler shaft placement (Fig. 3), and completion with one or two linear Endo-GIA™ firings (Covidien, Medtronic, USA) (Fig. 4). Stapler diameter (23 or 25 mm) was chosen based on proximal esophageal caliber. HS was standard until February 2022. Thereafter, an exclusive CS approach was adopted without the need for intraoperative conversion to manual suturing in any case.

All anastomoses were reinforced with an omental flap, leveraging its vascularity, immunomodulatory properties, and pro-regenerative mediators [9]. A Jackson-Pratt suction drain was routinely placed posterior to the anastomosis and maintained until oral intake. On postoperative day 7, contrast swallow study was routinely performed. In the absence of radiologic suspicion for leak, the nasogastric tube was removed and a soft diet initiated.

ALs were suspected based on clinical signs and/or radiologic findings and confirmed via contrast-enhanced CT scan as per Esophagectomy Complications Consensus Group (ECCG) criteria [10]. Anastomotic stricture was defined as dysphagia for which endoscopic dilation of the anastomosis was needed, excluding local recurrence [11].

### Postoperative surveillance

Structured follow-up included clinical evaluation and barium swallow at 3 months, and clinical exam, CT, and endoscopy at 6 months. Subsequent oncologic surveillance consisted of periodic endoscopy, CT (and tumor markers for adenocarcinomas) every 6–12 months, or earlier if indicated. Minimum follow-up for analysis was 3 months.

### Statistical analysis

Continuous variables were reported as mean ± standard deviation or median with interquartile range (IQR), categorical variables as counts with percentages. Normality of continuous variables was assessed using the Shapiro–Wilk test. Comparisons used Student’s t-test or Mann–Whitney U test for continuous variables, and chi-square or Fisher’s exact test for categorical variables. Two-sided p < 0.05 was considered significant. To minimize selection bias and effectively balance baseline confounders, Propensity Score Matching (PSM) was performed. Variables entered into the PSM model were selected a priori based on their established clinical relevance to anastomotic healing and the observed baseline disparities between cohorts. The model included: age, BMI, smoking status, tumor location, and type of neoadjuvant treatment. Matching was performed using a 1:1 nearest neighbor algorithm without replacement and a 0.3 caliper width to maximize the balance of matched pairs. A multivariable logistic regression model was constructed to identify independent predictors of AL. To ensure model stability and avoid overfitting given the number of events, variables were selected based on clinical significance and univariate signals: surgical technique, tumor location, and neoadjuvant therapy. Furthermore, a doubly robust estimation was performed by applying the multivariable model within the propensity-matched cohort as a sensitivity analysis. This combined approach was specifically implemented to provide a more stringent control for potential residual confounding and to confirm the statistical stability of our findings. To assess technical stability and evaluate potential learning-curve effects during the transition between the two techniques, a Cumulative Sum (CUSUM) analysis was performed with AL as the endpoint. Changepoint detection analysis was performed using the At Most One Change (AMOC) method. Analyses were performed in R 4.4.3 [12].

## Results

A total of 133 consecutive patients underwent McKeown esophagectomy during the study period: 127 were included in the final analysis. Of these, 67 received HS cervical anastomosis and 60 CS anastomosis. Baseline demographic and clinical characteristics are summarized in Table 1.

**Table 1:** Baseline characteristics of patients undergoing McKeown esophagectomy for esophageal cancer

	Before matching			After matching		
	Group HS	Group CS	P value	Group HS	Group CS	P value
Total	67 (100)	60 (100)		36 (100)	36 (100)	
Age, median (range)	65 (36–87)	72 (48–85)	**0.003**	69 (59–74)	70 (64–74)	0.367
Sex			0.972			0.814
Male	37 (55.2)	32 (53.3)		19 (52.8)	17 (47.2)	
Female	30 (44.8)	28 (46.7)		17 (47.2)	19 (52.8)	
BMI, median (range)	24 (14–35)	24 (16–38)	0.234	23 (21–27)	23.5 (22–25)	0.955
Smoking			**<0.001**			1
Non smokers	18 (26.9)	28 (46.7)		17 (47.2)	16 (44.4)	
Ex smokers	25 (37.3)	29 (48.3)		16 (44.4)	17 (47.2)	
Active Smokers	24 (35.8)	3 (5)		3 (8.3)	3 (8.3)	
Comorbidity			0.755			0.613
No	16 (23.9)	12 (20)		13 (36.1)	10 (27.8)	
Yes	51 (76.1)	48 (80)		23 (63.9)	26 (72.2)	
CCI, median (IQR)	5 (4–6)	5 (4–6)	0.128	5 (4–6)	5 (4–6)	0.729
ECOG PS			0.994			0.174
0	48 (71.6)	42 (70)		24 (66.7)	30 (83.3)	
1	19 (28.4)	18 (30)		12 (33.3)	6 (16.7)	
ASA score			0.075			0.798
I	7 (10.4)	4 (6.7)		4 (11.1)	4 (11.1)	
II	38 (56.7)	26 (43.3)		20 (55.6)	17 (47.2)	
III	22 (32.8)	30 (50)		12 (33.3)	15 (41.7)	
Preoperative albumin (g/L), median (IQR)	40 (39–44)	41 (41–43)	0.332	40 (38–43)	41 (38–43)	0.949
Preoperative histology			**<0.001**			0.540
Adenocarcinoma	6 (9)	23 (39.7)		5 (13.9)	8 (22.2)	
Squamous-cell carcinoma	61 (91)	34 (58.6)		31 (86.1)	28 (77.8)	
Undifferentiated	0 (0)	1 (1.7)		0 (0)	0 (0)	
Preoperative treatment			**<0.001**			0.602
Surgery alone	10 (14.9)	16 (26.7)		5 (13.9)	4 (11.1)	
Chemo-radiotherapy	55 (82.1)	32 (53.3)		29 (80.6)	27 (75)	
Chemotherapy	2 (3)	12 (20)		2 (5.6)	5 (13.9)	
Tumor location			**<0.001**			0.938
Cervical esophagus	13 (19.4)	2 (3.3)		1 (2.8)	2 (5.6)	
Upper esophagus	16 (23.9)	9 (15)		8 (22.2)	7 (19.4)	
Middle esophagus	32 (47.8)	23 (38.3)		21 (58.3)	19 (52.8)	
Distal esophagus	5 (7.5)	20 (33.3)		5 (13.9)	7 (19.4)	
EGJ Siewert I-II	1 (1.5)	6 (10)		1 (2.8)	1 (2.8)	
cT^a^			0.266			0.793
Tis/HGD	0 (0)	1 (1.7)		0 (0)	0 (0)	
T1a	1 (1.5)	3 (5)		0 (0)	0 (0)	
T1b	5 (7.5)	3 (5)		1 (2.8)	1 (2.8)	
T2	14 (20.9)	6 (10)		7 (19.4)	6 (16.7)	
T3	43 (64.2)	46 (76.7)		25 (69.4)	29 (80.6)	
T4a	3 (4.5)	1 (1.7)		1 (2.8)	0 (0)	
Tx	1 (1.5)	0 (0)		1 (2.8)	0 (0)	
cN^a^			0.168			0.580
N0	15 (22.4)	21 (35)		7 (19.4)	10 (27.8)	
N+	52 (77.6)	39 (65)		29 (80.6)	26 (72.2)	
cM^a^		0.668			1	
M0	65 (97)	58 (96.7)		34 (94.4)	35 (97.2)	
M+	2 (3)	2 (3.3)		2 (5.6)	1 (2.8)	

Values are n (%) except where otherwise indicated

^a^According to 8th edition AJCC/UICC staging

Before PSM, the CS group was older than the HS group (median age 65 vs. 72 years), reflecting either selection bias or a shift towards risk stratification beyond chronological age. Despite this, Eastern Cooperative Oncology Group (ECOG) performance status, American Society of Anesthesiology (ASA) class, and Charlson Comorbidity Index (CCI) did not differ significantly between groups [13]. Smoking status differed significantly, with more active smokers in the HS group (24/67, 35.8% vs. 3/60, 5%) [14].

Cervical and upper thoracic tumors and squamous histology predominated in the HS group, while distal thoracic and esophagogastric junction (EGJ) tumors and adenocarcinoma were more frequent in the CS group. Chemoradiotherapy was the most common neoadjuvant regimen in both groups but more frequent in HS (55/67, 82.1% vs. 32/60, 53.3%), while chemotherapy alone or upfront surgery were more frequent in CS.

Intraoperative blood loss was <200 mL in all patients. No laparoscopic conversions occurred, while thoracoscopic conversion was required in 2 HS and 1 CS cases. Pathological outcomes (R0 resection, stage, lymph node yield) were comparable (Table 2).

**Table 2:** Tumor characteristics details of patients undergoing McKeown esophagectomy for cancer

	Before matching			After matching		
	Group HS	Group CS	P value	Group HS	Group CS	P value
Histology (pathologic report)			**<0.001**			0.371
Adenocarcinoma	6 (9)	25 (41,7)		5 (13.9)	9 (25)	
Squamous-cell carcinoma	61 (91)	35 (58.3)		31 (86.1)	27 (75)	
Resection margin			0.246			0.493
R0	64 (95.5)	60 (100)		34 (94.4)	36 (100)	
R1- Circumferential	3 (4.5)	0 (0)		2 (5.6)	0 (0)	
pT^a^			0.449			0.943
pT0	29 (43.3)	19 (31.7)		15 (41.7)	17 (47.2)	
pTis/HGD	2 (3)	3 (5)		1 (2.8)	1 (2.8)	
pT1a	2 (3)	5 (8.3)		1 (2.8)	2 (5.6)	
pT1b	9 (13.4)	5 (8.3)		3 (8.3)	4 (11.1)	
pT2	9 (13.4)	9 (15)		6 (16.7)	4 (11.1)	
pT3	15 (22.4)	19 (31.7)		9 (25)	7 (19.4)	
pT4a	1 (1.5)	0 (0)		1 (2.8)	0 (0)	
pN^a^			0.967			0.514
pN0	49 (73.1)	42 (70)		24 (66.7)	29 (80.6)	
pN1	12 (17.9)	11 (18.3)		6 (16.7)	5 (13.9)	
pN2	4 (6)	4 (6.7)		4 (11.1)	1 (2.8)	
pN3	2 (3)	3 (5)		2 (5.6)	1 (2.8)	
Mandard Tumor Regression Grade			0.304			0.441
1	29 (50.9)	20 (45.5)		15 (41.7)	18 (50)	
2	7 (12.3)	8 (18.2)		4 (11.1)	6 (16.7)	
3	7 (12.3)	4 (9.1)		3 (8.3)	4 (11.1)	
4	10 (17.5)	12 (27.3)		6 (16.7)	4 (11.1)	
5	4 (7)	0 (0)		3 (8.3)	0 (0)	
Number of resected nodes, median (IQR)	31 (23–39)	34 (28–43)	0.054	28 (23–37)	34 (27–44)	0.073
Number of metastatic nodes, median (IQR)	0 (0–1)	0 (0–1)	0.952	0 (0–2)	0 (0–0)	**0.035**

Values are n (%) except where otherwise indicated

^a^According to 8th edition AJCC/UICC staging

Post-PSM two balanced cohorts of 36 patients each were obtained: overall complication rates did not differ (23/36, 63.9% vs. 21/36, 58.3%; *p* = 0.809), nor did major complications (6/36, 16.7% vs. 5/36, 13.9%, *p* = 1) (Table 3). ECCG-defined complications, intensive care unit (ICU) admission, ICU stay, mortality, and readmission were similar. Median hospital stay was shorter in CS (19 vs. 14 days, *p* < 0.001), with earlier resumption of oral feeding (Table 3). Anastomotic leak incidence was higher in HS (7/36, 19.4% vs. 3/36, 8.3%), though not statistically significant (*p* = 0.307; OR 2.63, 95% CI 0.63–11.2). All leaks were managed without surgery, mostly Type I leaks effectively drained through the pre-positioned surgical drain. In CS, 7 out of 8 leaks (87.5%) arose from the linear stapled closure (“blind pouch”) rather than from esophagogastric anastomosis on CT imaging, versus 5 out of 17 (29.4%) in HS (*p* = 0.011). After PSM, the same pattern persisted (1/7, 14.3% vs. 3/3, 100%, *p* = 0.033) [15]. Anastomotic strictures were more frequently observed in the HS group (11/36, 30.6% vs. 6/36, 16.7%), again without reaching statistical significance (*p* = 0.267; OR 2.20; 95% CI 0.71–6.79). The multivariable logistic regression model identified high tumor location as the sole independent predictor of anastomotic leak (OR 3.01; 95% CI 1.17–7.93; *p* = 0.023). The circular stapled technique did not achieve statistical independence, demonstrating only a non-significant protective trend (OR 0.56; 95% CI 0.19–1.57; *p* = 0.279). The doubly robust estimation corroborated these findings, confirming high tumor location as the primary driver of risk (*p* = 0.012) and validating the non-significant protective trend of the CS approach (*p* = 0.234). CUSUM analysis for anastomotic leaks (Online Resource 2 shows the graphs with results) illustrates the performance trends for the entire and matched cohorts, showing the distribution of events before and following the adoption of the circular stapled technique. Statistical changepoint analysis identified a significant shift in institutional performance at case 79 (February 2023).

**Table 3:** Postoperative complications of patients undergoing McKeown esophagectomy for cancer

	Before matching			After matching		
	Group HS	Group CS	P value	Group HS	Group CS	P value
Postoperative complication	56 (83.6)	36 (60)	**0.005**	23 (63.9)	21 (58.3)	0.809
ECCG Gastrointestinal						
Anastomotic leak	17 (25.4)	8 (13.3)	0.139	7 (19.4)	3 (8.3)	0.307
Anastomotic leak grading			1			0.475
Type I	14/17 (82.4)	7/8 (88)		4/7 (57.1)	3/3 (100)	
Type II	3/17 (17.6)	1/8 (12)		3/7 (42.9)	0 (0)	
Type III	0 (0)	0 (0)		0 (0)	0 (0)	
ECCG Pulmonary						
Respiratory failure	3 (4.5)	1 (1.7)	0.621	1 (2.8)	1 (2.8)	1
Pneumonia	8 (11.9)	4 (6)	0.478	3 (8.3)	2 (5.6)	1
Pleural effusion	1 (1.5)	4 (6)	0.188	0 (0)	1 (2.8)	1
Pneumothorax	2 (3)	2 (3.3)	1	2 (5.6)	2 (5.6)	1
ECCG Cardiac						
Dysrhythmia atrial	12 (17.9)	12 (20)	0.823	5 (13.9)	7 (19.4)	0.752
ECCG Infection						
Central venous line infection	3 (4.5)	2 (3.3)	1	0 (0)	2 (5.6)	0.493
Wound infection	3 (4.5)	2 (3.3)	1	3 (8.3)	1 (2.8)	0.614
Recurrent laryngeal nerve palsy						
Grade Ia	3 (4.5)	0 (0)	0.246	3 (8.3)	0 (0)	0.239
Grade IIIa	1 (1.5)	0 (0)	1	1 (2.8)	0 (0)	1
Clavien-Dindo score	56/67	36/60	0.495	23/36	21/36	0.564
I	1 (1.8)	1 (2.8)		0 (0)	1 (4.8)	
II	42 (75)	25 (69.4)		17 (73.9)	15 (71.4)	
IIIa	5 (8.9)	7 (19.4)		1 (4.3)	3 (14.3)	
IIIb	5 (8.9)	1 (2.8)		3 (13)	1 (4.8)	
IVa	3 (5.4)	2 (5.6)		2 (8.7)	1 (4.8)	
IVb	0 (0)	0 (0)		0 (0)	0 (0)	
V	0 (0)	0 (0)		0 (0)	0 (0)	
Clavien-Dindo score ≥IIIa	13 (19.4)	9 (15)	0.637	6 (16.7)	5 (13.9)	1
Clavien-Dindo score ≥IIIb	8 (13.3)	3 (5)	1	5 (13.9)	2 (5.6)	0.429
Postoperative ICU	49 (73.1)	33 (55)	**0.033**	24 (66.7)	18 (50)	0.232
ICU length of stay in days (IQR)	1 (1–1)	1 (1–1)	0.570	1 (1–1)	1 (1–1)	0.688
Days before resuming oral intake, median (IQR)	10 (9–15)	9 (8–9)	**<0.001**	10 (9–18)	9 (8–9)	**0.007**
Median LOS in days (IQR)	19 (15–25)	14 (12–17)	**<0.001**	19 (15–25)	14 (12–16)	**<0.001**
90-day readmission	2 (3)	1 (1.7)	1	2 (5.6)	0 (0)	1
30-day mortality	0 (0)	0 (0)		0 (0)	0 (0)	
90-day mortality	0 (0)	0 (0)		0 (0)	0 (0)	
Anastomotic stricture	20 (29.9)	12 (20)	0.284	11 (30.6)	6 (16.7)	0.267

Values are n (%) except where otherwise indicated

## Discussion

This propensity score-matched analysis evaluated the short-term clinical and functional outcomes following the transition from manual hand-sewn to mechanical circular stapled cervical anastomosis in minimally invasive McKeown esophagectomy. The institutional transition to CS technique, executed without subsequent reversion to manual suturing even in challenging surgical scenarios (post-chemoradiotherapy or high cervical dissection) serves as a methodological strength that enhances internal validity of the study. The decision to adopt the CS approach was primarily driven by the clinical objective of enhancing technical standardization. By implementing a mechanical method, we aimed to minimize operator-dependent variables and mitigate inter-surgeon performance variability. This pursuit of procedural uniformity is especially critical in high-volume and teaching centers to ensure reproducible safety profiles [16]. While the limited number of events warrants a cautious interpretation, the CUSUM analysis (Online Resource 2, graph A total cohort, graph B post-PSM) suggests a performance shift following the technical transition. Given the lead surgeon's extensive experience since establishing the program in 2009, the long-term hand-sewn experience had already reached a stable proficiency plateau. Notably, the absence of a post-transition failure spike demonstrates that the mechanical approach allowed for an immediate, safe implementation without a detrimental learning curve effect, validating technical standardization as a driver of predictable surgical outcomes.

In the present series, anastomotic leak and stricture rates were numerically lower in the CS cohort, although these differences did not reach statistical significance, indicating that both surgical techniques provide a comparable safety profile regarding major anastomotic complications. This lack of statistical significance closely reflects the results of our multivariable regression and doubly robust estimation, which demonstrated that the surgical technique is not an independent predictor of leakage. The observed numerical differences, while statistically similar, are clinically noteworthy when considering the morbidity of leaks and the burden of recurrent dilations. The safety of the CS approach was coupled with a significant improvement in short-term functional recovery parameters, evidenced by a shorter hospital stay and earlier resumption of oral intake. These findings suggest that while the mechanical approach does not independently alter the risk of dehiscence, its intrinsic procedural efficiency and reproducibility translate into tangible clinical benefits during the immediate postoperative phase.

Anastomotic integrity is notoriously multifactorial, depending on tissue perfusion, tension at anastomotic ends, patient-specific comorbidities and anatomical features, neoadjuvant therapy and technical aspects in the surgical gesture [17–19]. In our experience, the routine intraoperative implementation of ICG-FA to assess gastric conduit perfusion likely served as a baseline factor in mitigating ischemic complications across both group [20].

Available literature on the optimal cervical anastomotic technique remains highly conflicting. While some large randomized controlled trials have reported significantly lower leak and stricture rates with mechanical stapling, global meta-analyses frequently yield inconsistent results, with some showing a higher stricture risk for CS and others favoring hand-sewn approaches [21–28]. Heterogeneity across studies likely reflects variations in surgical technique (stapler size, suture method), conduit configuration, postoperative management (including nasogastric decompression, drain placement, feeding protocols, and timing of contrast studies), and inconsistent outcome definition. Reflecting this lack of surgical consensus, a recent comprehensive meta-analysis of 12 RCTs encompassing 2,015 patients demonstrated that stapled and hand-sewn cervical anastomoses yield thoroughly comparable short-term outcomes, with no significant differences in either leak (RR 1.30; 95% CI 0.87–1.92; *p* = 0.727) or stricture rates (RR 1.53; 95% CI 0.75–3.11; *p* = 0.451) [29]. Consequently, our findings reinforce the concept that the choice between HS and CS should be guided by institutional standardization goals and specific clinical scenarios, given that both methods achieve equivalent safety when properly executed.From a technical standpoint, HS allows for tailored suture placement, precise tension modulation, and optimal mucosal alignment. These features offer distinct advantages when managing fragile or irradiated tissues, or navigating narrowed esophageal lumens, while avoiding the circumferential metal staples that may reduce postoperative luminal distensibility and predispose to strictures [5]. Conversely, CS provides technical standardization and a reproducible caliber and its primary advantage in our matched cohorts was a significantly faster functional recovery and a shorter length of stay [30].

As surgery continue to evolve, it is essential to complement traditional safety metrics with an assessment of how different procedures affect key functional domains. Functional endpoints (e.g., dysphagia, reflux, swallowing quality) are gaining increasing recognition as crucial components for evaluating the overall quality of surgery [31]. Future research should seamlessly integrate comprehensive functional assessments to offer a more holistic understanding of the impact of surgical techniques on patient health and quality of life.

The primary limitations of this study are its retrospective, single-center design and the relatively small sample size, which inherently carry a risk of selection bias and limit the generalizability of the findings. This is reflected in the baseline differences regarding tumor location and neoadjuvant treatment observed before matching. Such disparities partly reflect the progressive institutional adoption of a three-stage approach even for tumors below the carina. This strategy is favored to mitigate the risk of severe complications in the event of an anastomotic leak, and to avoid one-lung ventilation in elderly or frail patients (as our two-stage approach typically involves a hybrid method for the thoracic anastomosis). Notably, most cervical leaks in our series were safely managed via the intraoperatively placed drain, with none requiring surgical re-exploration (Type III) [32, 33]. These baseline imbalances were addressed through PSM and multivariate regression models, enabling a more reliable comparison. Nonetheless, we acknowledge that our analysis, particularly following PSM (n=36 per group), is likely subject to a Type II (β) error, meaning it may be underpowered to detect statistically significant differences in primary outcomes. Based on the observed clinical effect (an 11.1% absolute risk reduction in AL), a post-hoc power analysis indicates that a sample size of approximately 148 patients per group would be required to achieve a statistical power of 80% (α=0.05). Given the relatively low incidence of esophageal cancer, reaching such a large cohort in a single Western center within a reasonable timeframe is challenging. Given the relatively low incidence of esophageal cancer, recruiting such a large cohort in a single Western center within a reasonable timeframe is challenging. In light of this, while we cannot claim statistical superiority regarding leak prevention, the safe implementation of the CS approach demonstrating a comparable complication profile to the traditional hand-sewn method supports its overall clinical value. Patient-reported outcome measures (PROMs) are currently being collected prospectively in our center, and these data will be instrumental in future evaluations to determine which anastomotic technique yields optimal long-term functional results.

## Conclusions

Circular stapled cervical anastomosis is a feasible and safe alternative to the hand-sewn technique, demonstrating a comparable clinical profile to hand-sewn reconstruction. While the limited sample size precludes a definitive claim of statistical superiority, our data suggest that the CS approach provides a reproducible technical model for standardizing cervical reconstruction. By potentially minimizing the technical variability inherent in manual suturing, this method offers a consistent strategy for Western practices seeking to standardize complex esophageal reconstructions. Future studies are needed to confirm these findings on a larger scale and to clarify the impact of different anastomotic techniques on functional outcomes, increasingly recognized as key indicators of surgical quality and patient well-being.

## Supplementary Information

Below is the link to the electronic supplementary material.

Supplementary Material 1.

Supplementary Material 2.

## Data Availability

The data that support the findings of this study are available from the corresponding author upon reasonable request.
